# Gender differences in adolescent sleep neurophysiology: a high-density sleep EEG study

**DOI:** 10.1038/s41598-020-72802-0

**Published:** 2020-09-28

**Authors:** Andjela Markovic, Michael Kaess, Leila Tarokh

**Affiliations:** 1 University Hospital of Child and Adolescent Psychiatry and Psychotherapy, University of Bern, Bolligenstrasse 111, Haus A, 3000 Bern, Switzerland; 2 Graduate School for Health Sciences, University of Bern, Bern, Switzerland; 3 Section for Translational Psychobiology in Child and Adolescent Psychiatry, Department of Child and Adolescent Psychiatry, Center for Psychosocial Medicine, University Hospital Heidelberg, Heidelberg, Germany

**Keywords:** Neuroscience, Circadian rhythms and sleep, Non-REM sleep, REM sleep

## Abstract

During adolescence, differences between males and females in physiology, behavior and risk for psychopathology are accentuated. The goal of the current study was to examine gender differences in sleep neurophysiology using high-density sleep EEG in early adolescence. We examined gender differences in sleep EEG power and coherence across frequency bands for both NREM and REM sleep in a sample of 61 adolescents (31 girls and 30 boys; mean age = 12.48; SD = 1.34). In addition, sleep spindles were individually detected and characterized. Compared to boys, girls had significantly greater spindle activity, as reflected in higher NREM sigma power, spindle amplitude, spindle frequency and spindle density over widespread regions. Furthermore, power in higher frequency bands (16.2–44 Hz) was larger in girls than boys in a state independent manner. Oscillatory activity across frequency bands and sleep states was generally more coherent in females as compared to males, suggesting greater connectivity in females. An exception to this finding was the alpha band during NREM and REM sleep, where coherence was higher (NREM) or not different (REM) in boys compared to girls. Sleep spindles are generated through thalamocortical circuits, and thus, the greater spindle activity across regions in females may represent a stronger thalamocortical circuit in adolescent females as compared to males. Moreover, greater global connectivity in females may reflect functional brain differences with implications for cognition and mental health. Given the pronounced gender differences, our study highlights the importance of taking gender into account when designing and interpreting studies of sleep neurophysiology.

## Introduction

A cascade of biological, behavioral and social changes characterizes adolescent development. These include the maturation of sleep physiology and behavior, which support healthy cognitive and emotional functioning. One of the most striking changes in sleep physiology across adolescence is an approximately 40% decline in low-frequency sleep electroencephalography (EEG) power (i.e. slow wave activity; SWA)^1–5^. The maturational decline in SWA takes place in a gender specific manner, occurring about one year earlier in girls than in boys (mean age of maximal decline: 12.53 years in girls and 13.74 years in boys) as demonstrated by Campbell et al.^6^ in a longitudinal study (n = 67). In this study, SWA at one EEG derivation (C3/A2 or C4/A1) was analyzed across ages spanning 9–18 years and factors influencing the timing of the decline were examined. Together sex and pubertal stage accounted for 67% of the between-subject variance in the timing of SWA decline. In a later longitudinal study spanning ages 12–21 years in a separate sample of adolescents^7^, not only did the decline in SWA commence earlier in girls than boys, but was steeper in boys as compared to girls.

The above-described changes to the sleep EEG during adolescent development parallel and likely reflect changes in cortical gray matter volume. Magnetic resonance imaging (MRI) studies of adolescent brain development report substantial anatomical maturation including a loss of gray matter volume^8^, increase of white matter volume^8–10^ and a reduction of cortical thickness^10^ during adolescence with gray matter and total brain volume reaching their peak size earlier in girls as compared to boys^11^. Indeed, two studies examining both sleep EEG power and MRI gray matter volume in the same sample found a correlation between slow wave activity and cortical gray matter volumes in adolescents^12,13^.

In addition to SWA, gender differences have also been reported for sleep spindles, transient oscillations between 11 and 16 Hz. Spindles are generated through thalamocortical loops^14^ and allow an opportunity to monitor the activity of the thalamocortical circuit. In adults, greater sleep spindle density has been reported for women as compared to men^15,16^ and linked to higher sleep stability in women^15^ and functional differences in the thalamocortical network between genders^16^. However, studies investigating developmental periods (e.g., adolescence) with a main focus on gender differences in sleep spindle activity are lacking. An exception to this is a large-scale study comprised of a few EEG derivations spanning the ages 4–97 years, which reported greater spindle density in females, but no differences for amplitude, duration or frequency of spindles^17^. Another recent study examining changes to sleep spindle activity in 134 individuals across the ages 12–21 years found greater frequency of fast spindles in girls as compared to boys, but no differences in amplitude, duration or density^18^. The discrepancy in findings from these studies may be related to the different distribution of age in their samples, since developmental factors may impact gender differences as previously discussed. Therefore, further investigations of gender differences in sleep spindle activity during developmental periods are necessary.

Though a number of studies have examined the influence of gender on the sleep EEG during adolescence, as described above, these studies only examined a few bands (e.g., SWA) or electrodes^6,7,19–21^. Given the strong topographic variation in power as a function of age^22^ and findings from a previous study showing region-dependent genetic influence on sleep EEG power in adolescence^23,24^, results from a single EEG derivation cannot be generalized to other derivations. To our knowledge, only one study has examined gender differences using high-density sleep EEG^25^. In this study of 11 boys and 11 girls ranging in age between 8.7 and 19.4 years, the authors observed higher SWA in girls over bilateral temporal regions, whereas boys had higher SWA over central and frontal regions. The generalizability of these findings is limited given the broad age range (> 10 years) and the large changes to the sleep EEG that occur during this period coupled with the modest sample size (i.e., 11 in each group) and the focus on SWA.

Therefore, the current study focused on gender differences in sleep neurophysiology using high-density sleep EEG power in the frequency range from 1 to 44 Hz in a sample of 61 adolescents (mean age = 12.5; SD = 1.3; 31 females) at two time points 6 months apart. Based on previous findings, we hypothesized that boys will show more sleep EEG power in lower frequencies due to the later developmental decline of SWA in males^6,7^. In line with previous findings in adults^15,16^ and adolescents^17,18^, we expected girls to demonstrate greater spindle activity. In addition to sleep EEG power, a measure largely influenced by the number of synchronously firing neurons, we examined sleep EEG coherence, an index of brain connectivity during sleep reflecting interactions between spatially segregated populations of neurons^26^. While a study in adults aged 17–69 years has shown differences between males and females in a measure of sleep EEG connectivity^27^, to the best of our knowledge no previous studies have examined gender differences in sleep EEG coherence in adolescence. Therefore, we based our hypothesis on EEG coherence studies in waking, expecting that girls will demonstrate increased coherence as compared to boys^28,29^.

## Methods

### Participants

Thirty-one girls (mean age = 12.13; SD = 1.67) and thirty boys (mean age = 12.83; SD = 0.75) between 9 and 14 years old (84% between 12 and 14 years) were recruited as part of a twin study examining the heritability of the sleep EEG^23,24,30,31^. There was no significant age difference between boys and girls. Participants were healthy and born after the 30th week of pregnancy. Pubertal status was assessed by means of a validated self-rating scale adapted from Petersen et al.^32^ which determines pubertal development via the appearance of secondary sexual characteristics. According to the taxonomy of Tanner^33^, pubertal development is divided into five stages ranging from stage 1 (preadolescent) to stage 5 (full sexual maturity). Due to the earlier timing of puberty in girls, and our recruitment based on age, pubertal status in our sample was significantly different (*p* < 0.001) between genders with girls (mean = 3; SD = 1) being on average one pubertal category ahead of boys (mean = 2; SD = 1). The study was performed according to the Declaration of Helsinki and approved by the responsible ethics committee of the Canton of Zurich. Written informed consent from parents and assent from participants were obtained.

### Procedures and EEG data analysis

Sleep EEG was recorded on two consecutive nights (adaptation and baseline) at two time points 6 months apart (Time 1 and Time 2). Forty-nine participants (22 females) completed both sleep EEG assessments (Time 1 and Time 2). The recordings were performed at families’ homes after at least five days of a fixed sleep schedule with 9.5–10 h of time in bed per night ensuring adequate sleep. Compliance to the sleep schedule was measured by means of actigraphy and sleep diaries. Data from the baseline night were used for analyses with the exception of three subjects at Time 1 and three subjects at Time 2 whose baseline night recordings were of insufficient quality and, therefore, data from the adaptation night were used. For two subjects, data from both nights at the first assessment had to be excluded from the analyses due to technical difficulties with the EEG equipment. Therefore, only data from the second assessment time point were available for these subjects.

All recordings were performed on a 64-channel Geodesics system (Electrical Geodesic Inc., Eugene, OR, USA). Two channels were used for electrooculogram, two for electromyogram and two for electrocardiogram, resulting in 58 EEG derivations. Data were acquired at 1000 Hz and downsampled to 250 Hz for analysis. Channels with poor quality of signal were excluded based on visual inspection of spectrograms. The signals at all derivations were recalculated to average reference after excluding bad channels. The recordings were scored in 30-s epochs according to standard criteria^34^. Power density spectra were calculated per epoch (5-s windows; Hanning window; no overlap) using MATLAB (Mathworks, Natick MA, USA). Epochs with artifacts were excluded via a semi-automatic procedure based on the moving average over 21 epochs when power exceeded a threshold in 0.8–4.6 Hz and 20–40 Hz ranges of frequencies as previously described in^35^. We examined EEG power at each derivation for all frequency bands and the two states—rapid eye movement (REM) and non-rapid eye movement (NREM) sleep. The average number of NREM sleep epochs (30 s per epoch) included in the analyses was 770 (SD = 88) for Time 1 and 743 (SD = 124) for Time 2, while the average number of REM sleep epochs was 277 (SD = 75) for Time 1 and 256 (SD = 62) for Time 2. The following frequency bands were analyzed: delta (1–4.6 Hz), theta (4.8–7.8 Hz), alpha (8–10.8 Hz), sigma (11–16 Hz), beta 1 (16.2–20 Hz), beta 2 (20.2–24 Hz), gamma 1 (24.2–34 Hz) and gamma 2 (34.2–44 Hz). In order to assess gender differences in the topographic distribution of power independent of absolute power differences, we also examined power at each derivation normalized by the total power across derivations.

In addition to band power, an algorithm based on the envelope of the bandpass filtered signal from the Hilbert transform as described by Rusterholz et al.^24^ was applied to detect individual spindles in the frequency range between 10 and 16 Hz and extract their main features including amplitude, frequency, duration and density. In addition to the entire spindle range (between 10 and 16 Hz), slow (between 10 and 12 Hz) and fast (between 12 and 16 Hz) spindles were analyzed separately, as differences in the topographic distribution of these two classes of spindles have been shown in adults^36^ suggesting different mechanisms of generation. In contrast to sigma power which we define as 11–16 Hz, we set the lower limit for the detection of individual spindle events at 10 Hz, because this allows us to detect spindles that are at the lower boundary.

Coherence was calculated between all possible channel pairs (i.e., 1653 connections) as $\frac{{\left|{P}_{xy}(f)\right|}^{2}}{{P}_{xx}(f){P}_{yy}(f)}$ where ${P}_{xy}(f)$ is the cross-spectral density and ${P}_{xx}(f)$ and ${P}_{yy}(f)$ are the auto-spectral density functions of the two signals^37^. Although a multitude of measures for connectivity have been developed^38^, we apply coherence as the most established and widely-used in EEG studies. In order to account for contamination through volume conduction, we included only electrode pairs with separations (i.e., arc length) between 10 and 20 cm in our analyses^39^ resulting in 559 connections.

### Statistical analysis

For each band and state, significance was determined using an ANOVA with factors Gender (Female, Male), Age (decimal number representing the exact age at Time 1 and Time 2), Pubertal Status (stages 1–5; representing pubertal development at Time 1 and Time 2), and Relatedness (29 twin pairs and one triplet coded as 30 categories) for both power and coherence. In other words, data from both time points (Time 1 and Time 2) were analyzed in a repeated measures design with Age and Pubertal Status as both between-subject and within-subject (Time 1 and Time 2) factors. The same model was used to determine statistical significance of the four spindle features (amplitude, frequency, duration and density). Since the phase of the menstrual cycle has been shown to affect sleep spindle activity^40,41^, we conducted the same statistical analysis in a subsample including only girls who have not started to menstruate with age matched boys (18 girls and 18 boys).

The same analysis (ANOVA with factors Gender, Age, Pubertal Status and Relatedness) was applied to sleep stage variables. ANOVAs were performed in R with the package *afex*. Because gender differences are the focus of this paper and previous research findings suggest an interaction between age and gender effects on sleep EEG power^6,7^, we limit our analysis to main effects and the analysis of the interactions in our model to gender by age interactions. For all sleep EEG parameters, effect sizes of gender differences were calculated by means of Cohen’s d^42^. All *p* values were corrected for multiple comparisons using the false discovery rate according to the Benjamini–Hochberg procedure^43^. In power and sleep spindle analysis, we corrected for the number of derivations (i.e., 58), while in the coherence analysis we corrected for the overall number of connections (i.e., 1653; all possible channel pairs).

## Results

### Gender effects

Participants showed sleep architecture typical for healthy adolescents of this age group sleeping an average of 9 h and exhibiting a sleep efficiency greater than 90% (Table 1). As revealed by the ANOVA (Table 1), boys had slightly more wake after sleep onset as compared to girls (*p* = 0.04).

**Table 1:** Sleep parameters for females and males.

Sleep parameter	Time 1		ANOVA		
	Female	Male	Gender	Age	Gender × age
Total sleep time (min)	537.00 (± 55.50)	528.00 (± 38.00)	1.13 (*p* = 0.29)	6.15 (*p* = **0.01**)	1.88 (*p* = 0.17)
Wake after sleep onset (min)	18.98 (± 18.91)	33.78 (± 32.85)	6.74 (*p* = **0.01**)	0.00 (*p* = 0.99)	0.00 (*p* = 0.97)
Sleep latency (min)	23.57 (± 18.60)	17.65 (± 10.13)	2.61 (*p* = 0.11)	3.96 (*p* = 0.05)	2.92 (*p* = 0.09)
Sleep efficiency (%)	92.52 (± 4.38)	90.91 (± 5.59)	2.31 (*p* = 0.13)	1.26 (*p* = 0.26)	0.88 (*p* = 0.35)
REM Latency (MIN)	98.98 (± 34.77)	109.88 (± 51.00)	2.65 (*p* = 0.11)	4.17 (*p* = **0.04**)	1.36 (*p* = 0.25)
Stage 2 (%)	47.40 (± 9.21)	41.89 (± 9.00)	3.06 (*p* = 0.08)	3.85 (*p* = 0.05)	0.00 (*p* = 0.94)
Slow wave sleep (%)	27.20 (± 8.46)	29.60 (± 9.30)	0.00 (*p* = 0.96)	6.87 (*p* = **0.01**)	0.37 (*p* = 0.54)
Stage REM (%)	24.71 (± 4.65)	27.99 (± 6.12)	1.44 (*p* = 0.23)	7.96 (*p* = **0.01**)	0.10 (*p* = 0.75)

Mean and standard deviation (in parentheses) of sleep parameters for females (n = 31) and males (n = 30) in our sample at first time point of assessment (Time 1; Time 2 not shown as there were no significant differences between the two time points). The percentages were calculated with respect to total sleep time. Sleep latency is defined as the first occurrence of stage 2 sleep following lights out. Results from our ANOVA with factors Gender (Female, Male), Age (continuous variable), Pubertal Status (stages 1–5), and Relatedness (29 twin pairs and one triplet coded as 30 categories) are reported for the factors Gender and Age as well as their interaction (F-values; *p* values in parentheses). The factor Relatedness only reached significance for sleep latency (F = 8.48; *p* = 0.005), while the factor Pubertal Status remained non-significant for all sleep parameters. *p *values were corrected for multiple comparisons by means of the false discovery rate and *p* values lower than 0.05 are shown in bold.

**Figure 1:**
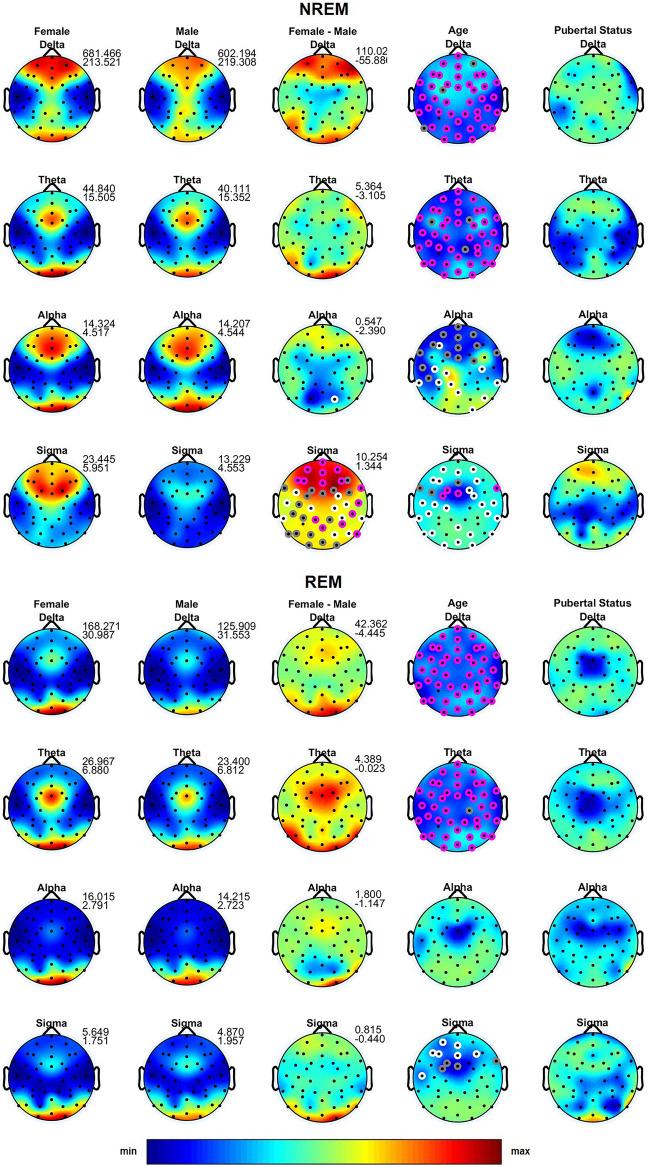
Topographic distribution of absolute sleep EEG power (µV^2^) across derivations for the delta to sigma bands during NREM and REM sleep. The first and the second column depict the values averaged across females and males on the same scale, while the third column depicts the difference between females and males. Minimum and maximum values are shown in the upper right corner of each topographic map. The fourth and the fifth column show the F-values from the analysis of variance (ANOVA) for the factors Age and Pubertal Status. In the third to fifth columns, significant electrodes are shown in white (*p* < 0.05), gray (*p* < 0.01) and magenta (*p* < 0.001). In these columns, warm colors represent increased activity in females, while cool colors represent increased activity in males. *p *values were corrected for multiple comparisons (i.e., the number of derivations) using the false discovery rate according to the Benjamini–Hochberg procedure.

**Figure 2:**
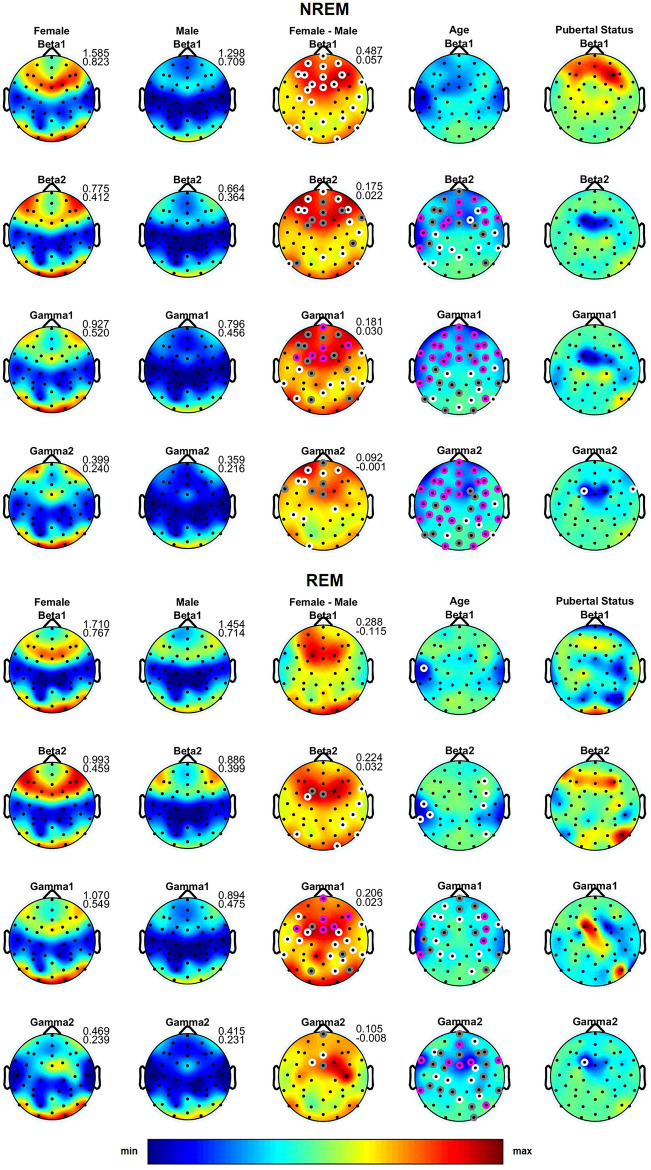
Topographic distribution of absolute sleep EEG power (µV^2^) across derivations for the beta 1 to gamma 2 bands during NREM and REM sleep. The first and the second column depict the values averaged across females and males on the same scale, while the third column depicts the difference between females and males. Minimum and maximum values are shown in the upper right corner of each topographic map. The fourth and the fifth column show the F-values from the analysis of variance (ANOVA) for the factors Age and Pubertal Status. In the third to fifth columns, significant electrodes are shown in white (*p* < 0.05), gray (*p* < 0.01) and magenta (*p* < 0.001). In these columns, warm colors represent increased activity in females, while cool colors represent increased activity in males. *p *values were corrected for multiple comparisons (i.e., the number of derivations) using the false discovery rate according to the Benjamini–Hochberg procedure.

Absolute sleep EEG power did not differ between boys and girls in the delta to alpha bands during NREM and REM sleep (Fig. 1; first and second column). In NREM, but not REM sleep, our ANOVA revealed a highly significant effect of gender in the sigma band, with girls showing greater power over all regions [Fig. 1; fourth row, third column; effect size = 0.51–0.92 with 86% of the significant derivations having an effect size between 0.5 and 0.8 (moderate) and another 14% having an effect size of at least 0.8 (large)]. Females also exhibited greater power in the beta and gamma bands in a state independent manner (Fig. 2; third column; effect size = 0.32–0.94; 71% moderate and 6% large effect size).

When sleep EEG power was normalized, gender differences were most pronounced in NREM delta (effect size = 0.27–0.94; 54% moderate and 11% large effect size) and sigma (effect size = 0.17–0.88; 61% moderate and 14% large effect size) bands with girls showing a more frontal focus and boys exhibiting a shift of maxima towards central/occipital regions in both of these frequency bands (Supplementary Figure 1). A similar pattern was also found in other frequency bands and both sleep states (Supplementary Figures 1 and 2).

**Figure 3:**
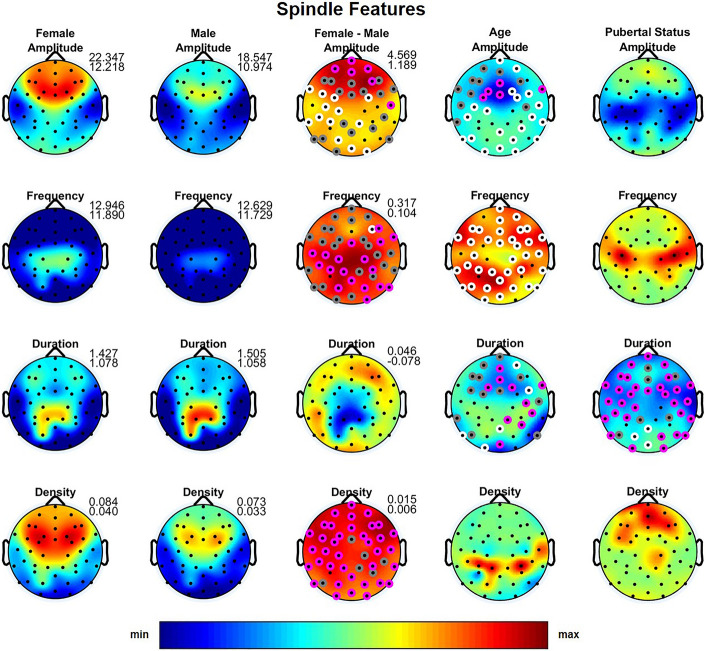
Topographic distribution across derivations for the four spindle features, i.e. amplitude (µV), frequency (Hz), duration (sec) and density (num/s). The first and the second column depict the values averaged across females and males on the same scale, while the third column depicts the difference between females and males. Minimum and maximum values are shown in the upper right corner of each topographic map. The fourth and the fifth column show the F-values from the analysis of variance (ANOVA) for the factors Age and Pubertal Status. In the third to fifth columns, significant electrodes are shown in white (*p* < 0.05), gray (*p* < 0.01) and magenta (*p* < 0.001). In these columns, warm colors represent increased activity in females, while cool colors represent increased activity in males. *p* values were corrected for multiple comparisons (i.e., the number of derivations) using the false discovery rate according to the Benjamini–Hochberg procedure.

With regards to sleep spindle features, we observed a highly significant effect of gender across derivations for spindle amplitude (effect size = 0.32–0.99; 57% moderate and 20% large effect size), frequency (effect size = 0.18–0.85; 72% moderate and 2% large effect size) and density (effect size = 0.29–1.04; 53% moderate and 33% large effect size) with girls showing higher amplitude, as well as denser and faster spindles (Fig. 3; third column). No such effect was found for spindle duration. These observations did not differ between slow and fast spindles. Performing the analysis in girls with no menarche and age matched boys yielded the same effects for slow spindles, but several differences in fast spindle features emerged: significantly longer spindle duration in girls, faster spindle frequency in boys and no significant gender differences in spindle density.

**Figure 4:**
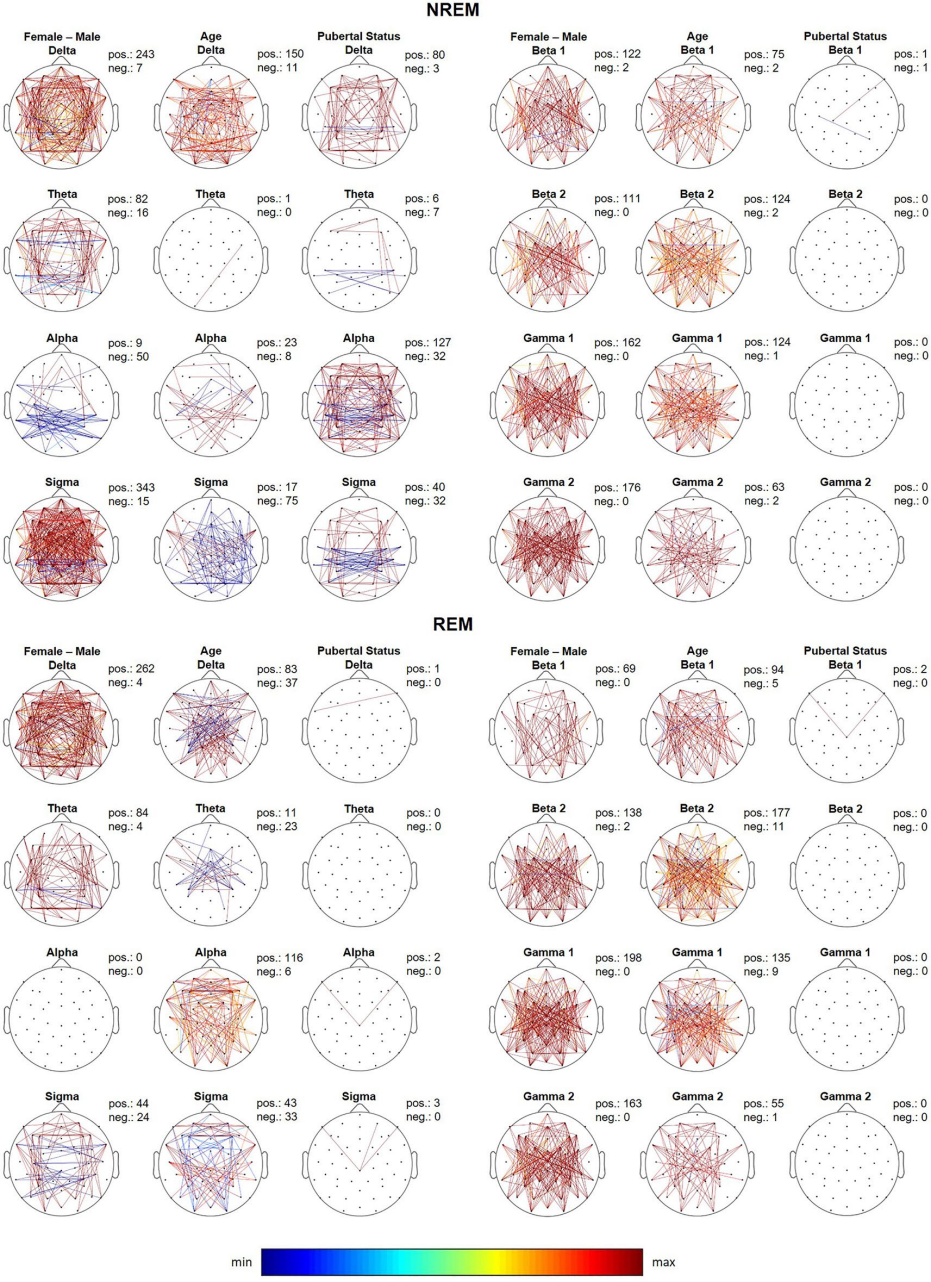
Topographic distribution of sleep EEG coherence across connections for all frequency bands during NREM and REM sleep. The first column depicts the difference between females and males. The second and the third column show the F-values from the analysis of variance (ANOVA) for the factors Age and Pubertal Status. Only connections with coherence values higher than 0.2 and *p* values lower than 0.05 are depicted. *p* values were corrected for multiple comparisons (i.e., the total number of connections) using the false discovery rate according to the Benjamini–Hochberg procedure. In order to make the distinction between positive and negative values clearer, the magnitude of each value was reduced by 30% resulting in a narrower range of values which amplifies the differences in color tones. Positive values depicted in warm colors represent increased activity in females, while negative values depicted in cool colors represent increased activity in males. The number of connections that showed increased coherence in females (pos) versus those that showed increased coherence in males (neg) is shown in the upper right corner of each topographic map. Similarly for age and pubertal status, connections which show a positive association with these factors (pos) are shown in warm colors while those that show a negative association (neg) are shown in cool colors and the number of such connections is shown in the upper right corner of each topographic map.

For sleep EEG coherence, we found the largest gender differences in the delta band for both NREM and REM sleep (effect size = 0.08–1.28; 51% moderate and 19% large effect size), and in the sigma band during NREM sleep (effect size = 0.12–1.26; 52% moderate and 22% large effect size) with girls showing greater coherence across the brain (Fig. 4; first column, left panel). Larger coherence values were found for girls in other bands and both sleep states as well, although the differences were less pronounced (Fig. 4; first column, right panel). The only exception was NREM alpha coherence (effect size = 0.32–0.99; 63% moderate and 7% large effect size), where boys had greater values focused over occipital and temporal regions (Fig. 4; first column, left panel). Furthermore, we found no significant gender differences for REM alpha coherence (Fig. 4; first column, left panel).

All significant gender effects including F- and *p*-values are listed in Supplementary Tables 1–4.

### Age effects

As expected, we found an age-related decline in total sleep time, slow wave sleep as well as a slight, but significant, increase in REM sleep and REM latency (Table 1). Furthermore, similar to previous studies^1–5^, we found an age-dependent decline in absolute EEG power across frequencies and sleep states (Figs. 1, 2; fourth column). Significant age effects on normalized NREM and REM sleep EEG power were found in the delta band, which demonstrated a topographic shift towards frontal regions with increasing age, while alpha, sigma, gamma 1 and gamma 2 (only in NREM sleep) bands showed an age-dependent shift towards posterior regions (Supplementary Figures 1 and 2). Shorter slow sleep spindle duration, lower spindle amplitude and higher spindle frequency were associated with older age (Fig. 3; fourth column). Coherence was significantly affected by age across bands and the two states with some connections showing a decrease but an overall increase in coherence with age (Fig. 4; second column).

### Pubertal effects

We found no significant effects of pubertal status on absolute (Figs. 1, 2; fifth column) and minimal effect on normalized (Supplementary Figures 1 and 2) sleep EEG power for any band or state. However, sleep spindle duration was affected by pubertal status with more advanced pubertal status being associated with shorter spindle duration (Fig. 3; fifth column). Pubertal status had a significant effect on NREM delta, alpha and sigma coherence with a number of connections over central and temporal regions demonstrating a decrease, but the majority of connections showing an increase with advancing pubertal status. We found no effect of pubertal status for the other frequency bands, REM sleep, or any other spindle features.

### Relatedness effects

Relatedness was significant for sleep latency (F = 8.48; *p* = 0.005), but no significant effects on any other parameter were observed.

### Gender by age interaction

Generally speaking, we found that boys start the decline in absolute sleep EEG power and sleep spindle features at a later age than girls. This was true of delta, theta, and alpha power during NREM sleep, beta 2, gamma 1 and gamma 2 power during REM sleep, and slow and fast spindle duration. There were no further significant gender by age interactions for power. In contrast, boys showed a decrease and girls showed an increase of spindle frequency with age; however, when all participants were pooled together a net increase was found. When examining coherence, we observed a significant interaction between age and gender for all frequencies, with the exception of theta and alpha, and both sleep states with boys experiencing the increase in coherence at older age as compared to girls. This interaction was regionally widespread but demonstrated a strong focus over central and temporal regions.

## Discussion

This study uses high-density sleep EEG to examine gender differences in sleep neurophysiology during early adolescence. Robust gender differences exist in the prevalence of psychiatric disorders^44–47^, many of which have their onset during adolescence, however, the neuroanatomical basis of these differences is not well understood^48^. Although the current literature supports gender differences in brain anatomy, differences are small and controversial^48^. Examining gender differences in the sleeping brain has several advantages. For one, sleep neurophysiology is a reflection of both structure and function making it more relevant to behavior. Furthermore, sleep, a time when the brain is “offline” and unaffected by implicit gendered expectations, may be the ideal time to examine gender differences in neurocircuitry. Our study also highlights the importance of taking gender differences into account in sleep studies.

### Sleep EEG power

We found a strong effect of gender for absolute sigma power and sleep spindles during NREM sleep, with girls exhibiting greater spindle amplitude, frequency and density across brain regions. Sleep spindles are oscillations in the sigma frequency range during NREM sleep, generated and synchronized by thalamocortical loops. Therefore, they reflect the function of this brain network. The greater NREM sigma activity observed in girls as compared to boys suggests gender differences in thalamocortical circuits. This observation is in line with sleep EEG studies in healthy adults which have shown greater spindle numbers and more sigma power in women as compared to men^15,16,49,50^. While we observe greater spindle amplitude, frequency and density in girls, one study of adolescents^18^ spanning the ages 12–21 years only found greater fast spindle density in girls and no difference in other spindle features. The discrepancy in findings may be due to the narrow age range in the current study which may provide more sensitivity to detect gender differences in sleep spindle activity, otherwise masked by the overall developmental pattern across a broader age range. Furthermore, the previous study assessed spindle activity at one frontal (F3; slow spindles) and one central (C3; fast spindles) derivation which limits their ability to detect local gender differences. Although previous studies have shown topographic differences between slow and fast spindles^36^ suggesting different generators, the gender differences we observed were consistent between the two spindle classes and widespread in topography.

The observed differences in sleep spindle activity between boys and girls may be of functional significance. For example, the well-established association between sleep spindle activity and memory consolidation/intelligence has been shown to differ between genders^50–52^. In an adolescent sample, Bódizs et al.^51^ found positive associations between spindle density as well as amplitude and fluid IQ in adult females, while in adult males greater fluid IQ was associated with higher spindle frequency. The authors suggest different cognitive strategies between males and females as a possible explanation.

Another possible functional implication of our findings relates to the role of sleep spindles in sleep protection^53,54^. Several empirical studies have shown that these oscillations can predict the ability to maintain sleep^55–57^. Dang-Vu et al.^56^ found that subjects demonstrating greater sleep spindle activity were more tolerant towards external sounds during sleep, i.e. they were able to stay asleep at higher levels of noise. Extrapolating from this, more spindle activity would suggest more protected sleep in females as compared to males. In support of this, we observed more wake after sleep onset in boys suggesting gender differences in sleep protection mechanisms. We note that this finding somewhat contradicts the current literature which reports higher rates of insomnia and more sleep difficulties in adult women. In fact, we find that subjective sleep quality as measured by the Sleep Habits Survey^58^ did not differ between boys and girls in our sample, and previous studies have suggested that gender differences in subjective sleep quality do not emerge till the end of puberty^59^. Finally, indirect evidence for gender differences in the thalamocortical system comes from waking studies which report gender differences in thalamocortical processing of auditory gating^60^ and pain perception^61^.

In addition to NREM sleep sigma power, girls in our sample exhibited greater absolute sleep EEG power in higher frequencies (beta 1 to gamma 2) for both NREM and REM sleep in line with several resting-state EEG studies in adults^62–64^. Because we find gender differences in high frequencies for both NREM and REM sleep, two states which are functionally and biologically unique^65^, we hypothesize that anatomical differences underlie the greater high-frequency power in females. As it is still unclear what brain structures are associated with beta and gamma power, future work should address this issue.

In contrast, the lower frequency bands (< 11 Hz) did not show differences in absolute sleep EEG power between boys and girls. The delta band has previously been shown to correlate with gray matter volume in the anterior cingulate and orbital frontal cortex^66^ and mirror the reduction of cortical gray matter volume in this age range^12,13^. As the age range in our sample is rather narrow and selected to reflect the period of most rapid changes in sleep EEG power^6^, gender differences in this frequency range may have been masked as boys start the pubertal decline of power later than girls. Therefore, our data set may constitute the last ascending part of the developmental trajectory in boys, while delta/theta power in girls is on the descending part of the maturational curve. This idea is supported by the interaction between the effects of gender and age we observed in the lower frequencies with power declining at older ages in boys as compared to girls. Furthermore, the large degree of inter-individual variability in the trajectories of both brain development^67^ and sleep EEG power^68^ further complicates this issue as some individuals may reach peak neuronal density/EEG power earlier than others. Examining developmental trajectories might, therefore, provide new insights into the nature of gender differences and should be taken into account in future studies.

For normalized sleep EEG power, we observed a topographic shift towards frontal regions in girls across frequencies and sleep states. Sleep studies in adults have shown attenuated differences between females and males when power is normalized suggesting that such differences are due to structural effects such as skull thickness^69^. However, our findings in adolescence do not provide support for this hypothesis. In our study, normalized power reveals additional differences related to topographic shifts previously masked by pronounced differences in absolute power. The topographic shift towards frontal regions in girls may be a reflection of advanced brain maturation according to maturational trajectories observed for topographic distribution of sleep EEG activity across childhood and adolescence^22^.

### Sleep EEG coherence

With regards to sleep EEG coherence, a measure of functional connectivity, we observed greater coherence in girls than boys across bands and states with the exception of NREM alpha coherence, where boys demonstrated higher values. These results are in agreement with previous waking EEG coherence studies in children and adolescents^28,29,70^. A recent sleep EEG study in adults found similar gender differences in the alpha and sigma bands during NREM sleep^27^ suggesting that these gender differences prevail over the life course. However, in the beta band the authors found the opposite gender effect with men exhibiting stronger connectivity than women during both NREM and REM sleep. We conjecture that it might be developmental changes that underlie the differences between this study and our results. Larger coherence values in girls have previously been hypothesized to reflect a maturational lead in females^70^. Interestingly, we find the largest differences between males and females in NREM delta and sigma power, two oscillations which rely on corticocortical and thalamocortical connectivity^14,71–74^. Both greater anatomical connectivity^75^ as well as greater functional connectivity^76,77^ have been observed for women in MRI studies. In line with our findings, a large MRI study of adult subjects found not only higher functional connectivity in women than in men, but also that gender differences were maximal in the anterior thalamus^77^. The authors suggest that greater connectivity in women may facilitate functions relying on synchronization between wide brain areas and emphasize the implication of such findings for psychiatric disorders associated with altered brain connectivity^77^. Taken together, gender differences in sleep EEG parameters vary between frequency bands^78,79^ and are therefore more likely to reflect functional differences in brain networks^16^ than merely differences in brain structure.

### Impact of age and puberty

Age had a significant influence on power and coherence across frequency bands and sleep states, which is likely due to anatomical changes to the brain^8,80^. On the other hand, we did not find an effect of pubertal status (with the exception of low-frequency coherence during NREM sleep). This finding is in agreement with results from a longitudinal study by Feinberg et al.^20^ showing that the association between the rate of the NREM delta power decline and the rate of pubertal development disappears when controlling for age. The authors hypothesize that the power decline is associated with developmental processes other than physical or sexual maturation. Further evidence for this notion comes from a study investigating delta power in girls with central precocious puberty^81^ concluding that increased estrogen does not cause the adolescent decline in delta power.

### Limitations

Several limitations are associated with the current study. The analyzed data originates from a twin study examining heritability of the sleep EEG during adolescence^24^. Therefore, our sample does not fully meet the criterion for data independence. However, in order to account for this limitation, we included relatedness as a factor in our analyses and found that this factor is not a significant contributor. In addition, we performed all our analyses with half of the sample including only those participants who were not related to each other (15 girls and 15 boys). Performing analyses in this way, we obtained the same results as observed when including the entire sample only less pronounced, most likely due to lower statistical power associated with the smaller sample size.

A further limitation is that we were not able to control for the menstrual cycle, the phase of which can have an influence on sleep spindle activity due to hormonal variability^40,41^. However, as thirteen girls (42%) in our sample reported already having started to menstruate, we were able to compare spindle features in girls with no menarche and age matched boys. Performing the analysis in this way does not impact our findings suggesting that the observed gender differences are stable despite variability introduced by the presence of menstruation in approximately half the sample. The only exception to note was a difference in the direction of effect for fast spindle frequency with boys demonstrating faster spindles as compared to girls with no menarche, whereas their spindle frequency was decreased as compared to girls when we included the whole sample. Also, we found significantly longer fast spindle duration in girls with no menarche and no significant gender differences in fast spindle density. Therefore, we believe that our findings are representative and generalizable and as such add to the current body of research on gender differences in sleep EEG during adolescence.

We cannot rule out the possibility that our results for EEG coherence have been affected by volume conduction. In order to reduce such effects, we only included pairs of electrodes with separations between 10 and 20 cm as previously recommended^39^. Furthermore, we used average reference suggested to provide the best approximation of absolute potentials for high-density EEG data^82^. The decision to use coherence as our measure of connectivity was based on the fact that the majority of EEG studies use this metric. As our findings are in agreement with previous work from both MRI^76,77^ and EEG^27–29,70^ studies, we believe that volume conduction and the choice of method do not significantly impact our results.

Finally, we note that we refer to gender instead of sex, as we did not assess biological sex nor the secondary sexual characteristics of our participants. The distinction between the groups was based on their own perception, which is by convention termed gender.

## Conclusion

To summarize, our study demonstrates gender differences with moderate to large effect sizes in sleep EEG power and coherence most pronounced in the sigma band during NREM sleep. While some of the gender differences we report could be influenced by anatomical measures, it is highly unlikely that brain structure is the only driving force of such differences, as we show strong effects for specific oscillations. This paper adds to current efforts to unravel the nature of differences between the genders with broad implications for psychiatry, neuroscience and neurology, as the presentation and the course of disorders vary between genders^83^. As previous studies have shown that gender differences in the sleep EEG vary under different experimental conditions and states such as health and illness^78,79^, such differences are, therefore, more likely to reflect functional differences in the generating brain networks^16^. Although we observe robust differences between males and females in our sample, we cannot infer differences in behaviour and functioning between the genders. Despite being subtler than previously thought, gender differences are important to understand for prevention and therapy in most branches of medicine including psychiatry.

## Supplementary information

Supplementary Figure 1.

Supplementary Figure 2.

Supplementary Table 1.

Supplementary Table 2.

Supplementary Table 3.

Supplementary Table 4.

## Data Availability

The datasets generated and analyzed during the current study are available from the corresponding author on reasonable request and pending ethics approval.
