# Supplementary material for: Gender differences in adolescent sleep neurophysiology: a high-density sleep EEG study
Source: Sci Rep. 2020 Sep 28;10:15935. doi: 10.1038/s41598-020-72802-0 (PMC7522718; doi:10.1038/s41598-020-72802-0)
Supplement: Supplementary file 1 — Supplementary Figure 1. [file 41598_2020_72802_MOESM1_ESM.docx]

**Gender Differences in Adolescent Sleep Neurophysiology: A High-Density Sleep EEG Study**

Andjela Markovic^1,2^, Michael Kaess^1,3^, Leila Tarokh^1^

^1^University Hospital of Child and Adolescent Psychiatry and Psychotherapy, University of Bern, Bern, Switzerland

^2^Graduate School for Health Sciences, University of Bern, Bern, Switzerland

^3^Section for Translational Psychobiology in Child and Adolescent Psychiatry, Department of Child and Adolescent Psychiatry, Center for Psychosocial Medicine, University Hospital Heidelberg, Heidelberg, Germany


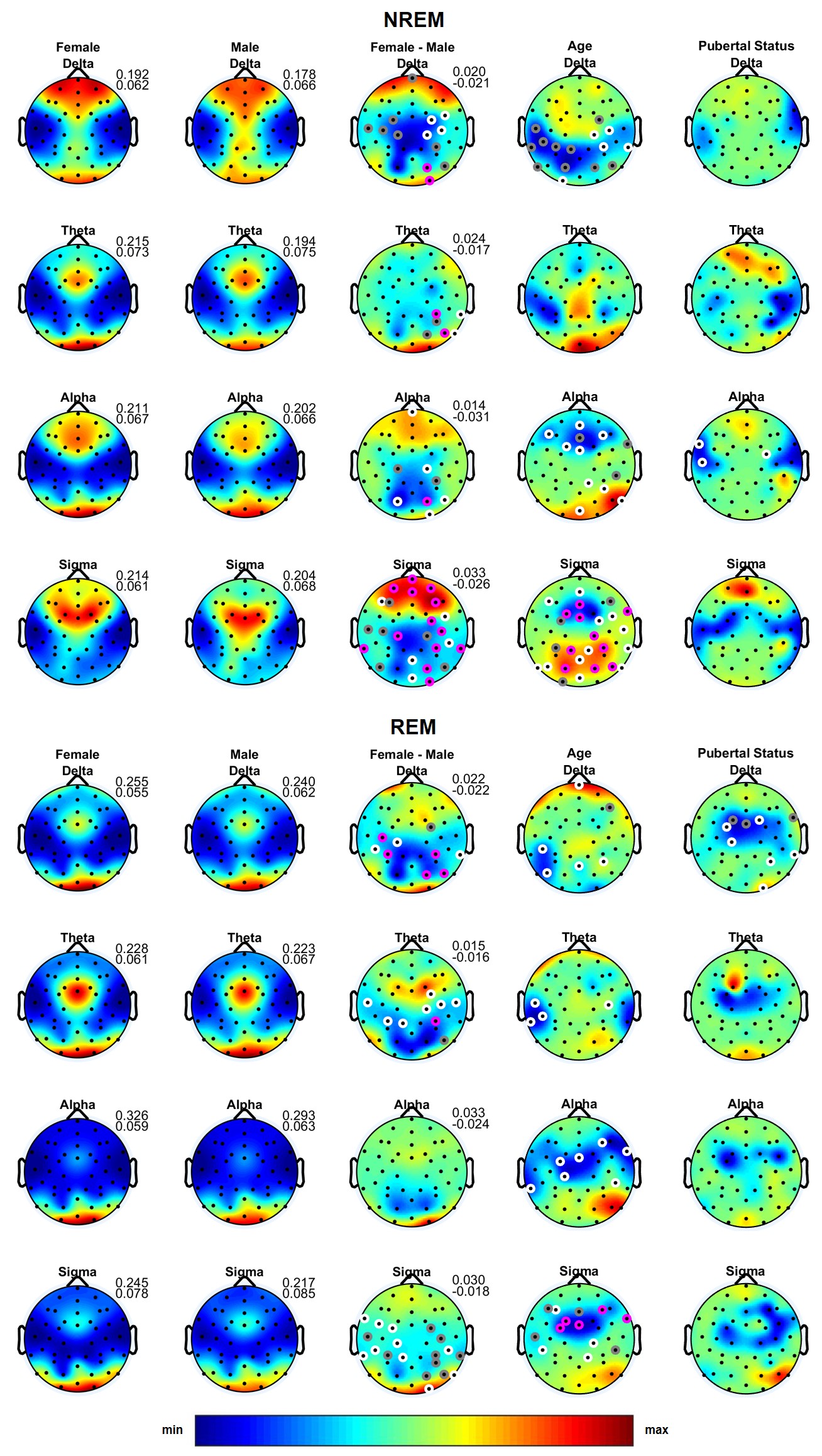


Supplementary Figure 1: Topographic distribution of sleep EEG power (µV^2^) normalized at each derivation by the total power across derivations for the delta to sigma bands during NREM and REM sleep. The first and the second column depict the values averaged across females and males on the same scale, while the third column depicts the difference between females and males. Minimum and maximum values are shown in the upper right corner of each topographic map. The fourth and the fifth column show the F-values from the analysis of variance (ANOVA) for the factors Age and Pubertal Status. In the third to fifth columns, significant electrodes are shown in white (p < 0.05), gray (p < 0.01) and magenta (p < 0.001). In these columns, warm colors represent increased activity in females, while cool colors represent increased activity in males. P-values were corrected for multiple comparisons (i.e., the number of derivations) using the false discovery rate according to the Benjamini-Hochberg procedure.
