# Supplementary material for: Gender differences in adolescent sleep neurophysiology: a high-density sleep EEG study
Source: Sci Rep. 2020 Sep 28;10:15935. doi: 10.1038/s41598-020-72802-0 (PMC7522718; doi:10.1038/s41598-020-72802-0)
Supplement: Supplementary file 3 — Supplementary Table 1. [file 41598_2020_72802_MOESM3_ESM.docx]

**Gender Differences in Adolescent Sleep Neurophysiology: A High-Density Sleep EEG Study**

Andjela Markovic^1,2^, Michael Kaess^1,3^, Leila Tarokh^1^

^1^University Hospital of Child and Adolescent Psychiatry and Psychotherapy, University of Bern, Bern, Switzerland

^2^Graduate School for Health Sciences, University of Bern, Bern, Switzerland

^3^Section for Translational Psychobiology in Child and Adolescent Psychiatry, Department of Child and Adolescent Psychiatry, Center for Psychosocial Medicine, University Hospital Heidelberg, Heidelberg, Germany

**Supplementary Table 1**: Significant gender differences in absolute sleep EEG power for all frequency bands and both sleep states

| **Frequency Band** | **NREM** | | **REM** | |
| --- | --- | --- | --- | --- |
|  | Female-Male | F-value  p-value (number of significant derivations) | Female-Male | F-value  p-value (number of significant derivations) |
| Alpha | -1.19 | F=10.56  p=0.048 (1) | / | / |
| Sigma | 1.34 to 10.22 | 4.41≤F≤18.19  0.001<p≤0.041 (43) | / | / |
| Beta 1 | 0.16 to 0.49 | 4.97≤F≤14.03  0.016≤p≤0.049 (24) | / | / |
| Beta 2 | 0.07 to 0.15 | 5.40≤F≤15.63  0.004≤p≤0.043 (21) | 0.08 to 0.22 | 7.19≤F≤18.2  0.003≤p≤0.046 (10) |
| Gamma 1 | 0.06 to 0.18 | 5.37≤F≤21.87  0.001<p≤0.04 (26) | 0.08 to 0.21 | 5.26≤F≤27.51  0.001<p≤0.047 (24) |
| Gamma 2 | 0.03 to 0.09 | 6.32≤F≤18.01  0.001≤p≤0.047 (14) | 0.04 to 0.07 | 11.37≤F≤17.27  0.004≤p≤0.016 (4) |

Only significant results after correction for multiple comparisons (i.e., the number of derivations) using the false discovery rate according to the Benjamini-Hochberg procedure are listed. The first column shows the frequency band. For NREM sleep, the second column shows the range of significant differences (Female-Male), while the corresponding range of F-values as well as p-values from the analysis of variance (ANOVA) is shown in the third column (the number of significant derivations is depicted in parentheses). Results for REM sleep are shown in columns four and five.
